# Perceptions of intercultural competence and institutional intercultural inclusiveness among first year medical students: a 4-year study

**DOI:** 10.1186/s12909-019-1780-y

**Published:** 2019-09-11

**Authors:** Bunmi S. Malau-Aduli, Simone Ross, Mary D. Adu

**Affiliations:** College of Medicine and Dentistry, Division of Tropical Health and Medicine, James Cook University, QLD, Townsville, 4811 Australia

**Keywords:** Medical students, Intercultural competence, Institutional intercultural inclusiveness

## Abstract

**Background:**

This study sought to examine the awareness/perception of intercultural competence and institutional intercultural inclusiveness among first year students at an Australian medical school over four consecutive years (2014–2017); to identify existing gaps in the curriculum and proffer recommendations.

**Methods:**

The study employed an adapted 20-item questionnaire for data collection. The reliability and interrelations of the survey items were examined. Descriptive statistics was used to examine students’ perceptions, while Mann-U Whitney and Kruskal-Wallis tests were used to assess items scores in relation to participant characteristics.

**Results:**

Over the 4 years of study, there were 520 respondents with between 53 to 69% response rates per year. Cronbach’s alpha for the instrument was 0.88 and factor analysis showed all items loading strongly on two components. Participants’ mean score on self-reported intercultural competence levels ranged from 3.8–4.6 out of 5; indicating relatively high awareness, valuing and understanding of cultural differences among this group of students. However, their mean scores (3.4–4.2) for institutional intercultural inclusiveness were slightly lower.

**Conclusion:**

The instrument used in this study is effective in assessing level of intercultural competence among medical students. However, the results highlight the need for increased institutional support and professional development for faculty members to foster institutional intercultural inclusiveness.

## Background

The push for internationalisation of Australian Higher Education (HE) is evident in the increasing international student population and ethnic diversity in classrooms and campuses across Australia [1]. In view of this, intercultural competence has become essential in all occupations and cultural settings [2]. Byram (1997, p34) defined intercultural competence as “Knowledge of others; knowledge of self; skills to interpret and relate; skills to discover and/or to interact; valuing others’ values, beliefs, and behaviors; and relativizing one’s self” [3]. To effectively interact with people from other cultures, one must be interested, be sensitive enough to perceive cultural differences and be inclined to adapt to new situations and respect other people’s worldviews [4].

Many professional courses require graduates to be able to work in culturally diverse contexts. The Australian Medical Council (AMC) emphasises the responsibility of medical schools to ensure that intercultural competence underpins the training and professional development of their trainees [5]. Medical educators and accreditation bodies recognise intercultural competence as critical to the professional development of medical students [6]. Intercultural competence refers to a set of congruent behaviour, attitudes and policies that come together in a system, agency or health service, or among professionals, which enables the organisation or the professionals to work effectively in cross cultural settings [7]. Intercultural competence in medical practice is an important strategy to address ethnic inequalities in quality of care [8, 9]. Intercultural competence enhances care providers’ ability to recognise and respond to the healthcare needs, values, beliefs and behaviours of patients from ethnic minority groups [10] and it is a major determinant of patients’ health outcomes [11].

Education is the indispensable tool by which society ensures the learning of behaviours and values necessary to sustain culture and to encourage cultural awareness [4]. Therefore, incorporating intercultural competence training into medical programs is important due to two major factors. Firstly, intercultural competence ensures excellent provider-patient communication and helps eliminate racial/ethnic disparities in medical care [12, 13]. Secondly, it improves knowledge, understanding and skills for meeting health needs of the growing diverse population [14–16]. Thus, it prepares students to care for patients from diverse social and cultural backgrounds, to recognise and appropriately address cultural and gender biases in themselves and in the line of health care delivery to ensure positive health outcomes [17, 18].

The concept of intercultural competence has its foundation based in cross-cultural training. Acquisition of intercultural competence in medical education can be divided into 3 major domains namely: 1) Cognitive - knowledge, 2) Affective - attitude, values and biases, and 3) Skills [19]. Demonstration of intercultural competence means all three domains are used to overcome barriers to achieving quality cross-cultural care with patients [20]. The cognitive component of the model relates to self-awareness and knowledge that culture exists; which has been recognised as the first crucial aspect in intercultural competence development because it assists an individual to attune appropriate behavioral adjustment to cultural differences [21, 22]. Additionally, individual characteristics (such as age, gender etc.) have been tagged as predictors of intercultural competence development [23] because different experiences will have an impact on a person’s ability for cross-cultural adaptation [24]. Adequate and objective assessment of the external outcomes (skills) in medical education requires practical demonstration through cross-cultural interactions with volunteer patients, structured clinical exams or clinical presentation while on placement, whereas knowledge and attitude (internal outcomes) can be assessed through the use of standard survey tools [25].

Attitude which involves consciousness of one’s own intercultural competence and an internal shift in frame of reference, is a fundamental starting point of process orientation and it consequently enhances the external observable (behaving and communicating appropriately and effectively in intercultural situations) outcomes [3, 26]. The foundational role of attitude in intercultural competence was emphasised in the following statement by Okayama, Furoto and Edmonson (2001: p97)

*“to maintain culturally competent attitudes as we continue to attain new knowledge and skills while building new relationships. Awareness, the valuing of all cultures and a willingness to make changes are underlying attitudes that support everything that can be taught or learned”* [27].

Therefore assessing first year medical students’ consciousness of their own intercultural competence could stimulate the students’ interest in developing capability to adapt to new cultural contexts for effective cross-cultural interactions with patients from different ethnic backgrounds as they progress in their medical training and career path. Additionally, such assessment may aid early identification of learning gaps that could subsequently facilitate the establishment of training strategies that enhance the curriculum and foster professional development.

Furthermore, institutional intercultural inclusiveness is another essential facilitator of intercultural competence among students. This is because it creates an environment where the learning needs of all students from diverse cultures are met and individual students are able to participate fully in classrooms, with better academic achievements and enhanced career prospects [28]. Inclusive learning environments improve students’ ability to communicate and work in cultural diverse settings, enhance their social self-efficacy and intercultural competence outcomes [29].

Therefore, our study examined first year medical students’ consciousness of their own intercultural competence levels and their perceptions of intercultural inclusiveness in the institution. This evaluation process was aimed at fostering awareness among the students of their own attitude, values and biases. It also aided faculty in identifying areas of teaching that are in need of improvement. The study also cross-validated the cultural competence survey tool used and assessed its reliability in measuring intercultural competence levels.

## Methodology

### Institutional context

Intercultural competence is emphasised in the James Cook University’s (JCU) Graduate Attributes Statement, which states that “JCU graduates are committed to reconciliation, diversity, and sustainability”. There is therefore a need to create a culturally sensitive practice that contributes to a responsible pedagogy that empowers students to be culturally competent. The JCU medical school has a small group learning program called ‘Home Group’ for years 1 to 3 students, that has an aim of transitioning year one students to the medical degree and University life [30]. Each group is designed to have a mix of gender, nationality, and age. In all six semesters of years 1 to 3 (2 semesters in each year of study), there is a compulsory social medicine subject. In year 1 there are activities designed for students to learn about each other and their backgrounds, including culture, language and healthcare belief. Both semesters in year 1 provide learning about culture; however, the second semester of year 1 has detailed learning about culture and the relevance of intercultural competence in medicine. The learning activities focus on awareness of one’s own and other people’s worldviews; checking assumptions and being open to diversity; respectful inquisitiveness in caring for individuals, being non-judgmental and sensitive to diverse groups, language and the challenging factors for healthcare; communicating appropriately across cultures; accessing an interpreter and the effects of racism on social and physical health.

### Study procedure and participants

The study was conducted over a 4-year period (2014–2017) and all first year medical students in each year were invited to complete the questionnaire at the start of the second academic study period. The study received ethics approval from the James Cook University Human Research Ethics Committee (H5817).

### Survey instrument

The survey instrument contained items on the socio-demographic characteristics of the students; 13 items on self-reported intercultural competence and 7 items on satisfaction with the institutional approach to intercultural inclusiveness. The survey items were adapted from Mak et.al’s [31] study on cultural inclusiveness and students’ cultural learning. The instrument was initially used at the University of Tasmania and permission was sought for use at JCU. The response format for each of the instrument items was a 5-point Likert scale, with 1 being “strongly disagree” and 5 being “strongly agree”. There were no negatively worded statements, so the higher the score, the stronger the perception on intercultural competence and institutional inclusiveness. The highest mean score possible was five.

### Data collection and analysis

Paper based questionnaires were administered to respondents in classroom settings and collated immediately upon completion. The collected data was captured with REMARK, transferred into Microsoft excel and analysed using IBM SPSS (V 23). Data was checked for completeness before analysis and the proportion of missing data for any of the items did not exceed 1.5%.

Internal reliability of the instrument was calculated using Cronbach’s alpha test [32]. Principal factor analysis was used to ascertain the interrelations between the items in the instrument and the extent to which the items form the underlying themes [33].

Mean scores were calculated for responses to each of the items, while non-parametric (Mann-U Whitney and Kruskal-Wallis) tests were used to determine if any of the socio-demographic variables (age, gender, time spent in Australia, ethnic origin and rurality) influenced participants’ responses. Non-parametric tests were used because the data was not normally distributed. Mann-U Whitney test was used for variables with two independent groups, Kruskal-Wallis test was used for variables that had more than two groups. Statistical significance was set at *p* < 0.01.

## Results

### Description of respondent sample

Table 1 shows the distribution of the students based on the academic calendar years 2014–2017. Of the 520 respondents, there were almost twice as many females (62.1%) as males (33.8%). Majority of the participants were below the age of 20 years (72.3%), were of Australian descent (56.5%), and had lived in a major city (38.3%) prior to attending university.

**Table 1:** Demographic profile of participants

Characteristics	Frequency of participants within each year of study (%)				
	2014 (*n* = 137)	2015 (*n* = 105)	2016 (*n* = 141)	2017 (n = 137)	Overall (*N* = 520)
Gender					
*Male*	34.5	40.0	27.6	40.1	33.8
*Female*	65.5	60.0	72.4	59.9	62.2
Age group (years)					
*≤ 20*	74.9	74.3	74.3	65.5	72.3
*> 20*	25.1	25.9	25.7	34.5	27.7
Ethnic Origin					
*Africa*	9.5	11.5	6.4	4.5	7.7
*Asia*	29.2	22.1	26.4	26.5	26.0
*Australia*	52.6	58.7	59.3	59.1	56.5
*Europe*	3.6	4.8	5.0	3.8	4.2
*America*	5.1	2.9	2.8	6.1	4.2
Rurality/Town					
*Rural town*	19.7	25.6	34.3	34.0	31.0
*Regional centre*	43.3	35.6	28.3	23.0	29.6
*Major city*	35.8	38.5	37.7	43.0	38.3
Time lived in Australia					
*< 1*	2.2	11.4	10.6	11.7	14.0
*1–5 years*	3.6	8.6	7.8	10.9	7.7
*6–10 years*	10.2	8.6	10.6	9.5	9.8
*11–15 years*	10.9	8.6	4.3	5.1	7.3
*> 15*	3.6	3.8	8.5	5.8	5.6
*Born in Australia*	49.6	59.0	58.2	56.2	55.6

### Reliability of survey instrument

The instrument was reliable and demonstrates an acceptable level of internal consistency with a Cronbach alpha of 0.89 for the self-reported intercultural competence scale (13 items), 0.85 for the institutional intercultural inclusiveness scale (7 items), and 0.88 for the combined 20 items.

### Factor analysis

Principal component analysis was the form of Factor Analysis employed in this study. The strength of inter-correlation between the items was confirmed using Kaiser-Melyer Olkin and Bartlett’s test of sphericity [33]. The Kaiser-Meyer-Olkin score for these set of items was 0.87 and the Bartlett’s test of sphericity was significant (*p* = 0.000); these indicate that the data sets are adequate and suitable for factor analysis. Correlation matrix revealed that all items have a coefficient of above 0.3.

**Fig. 1:**
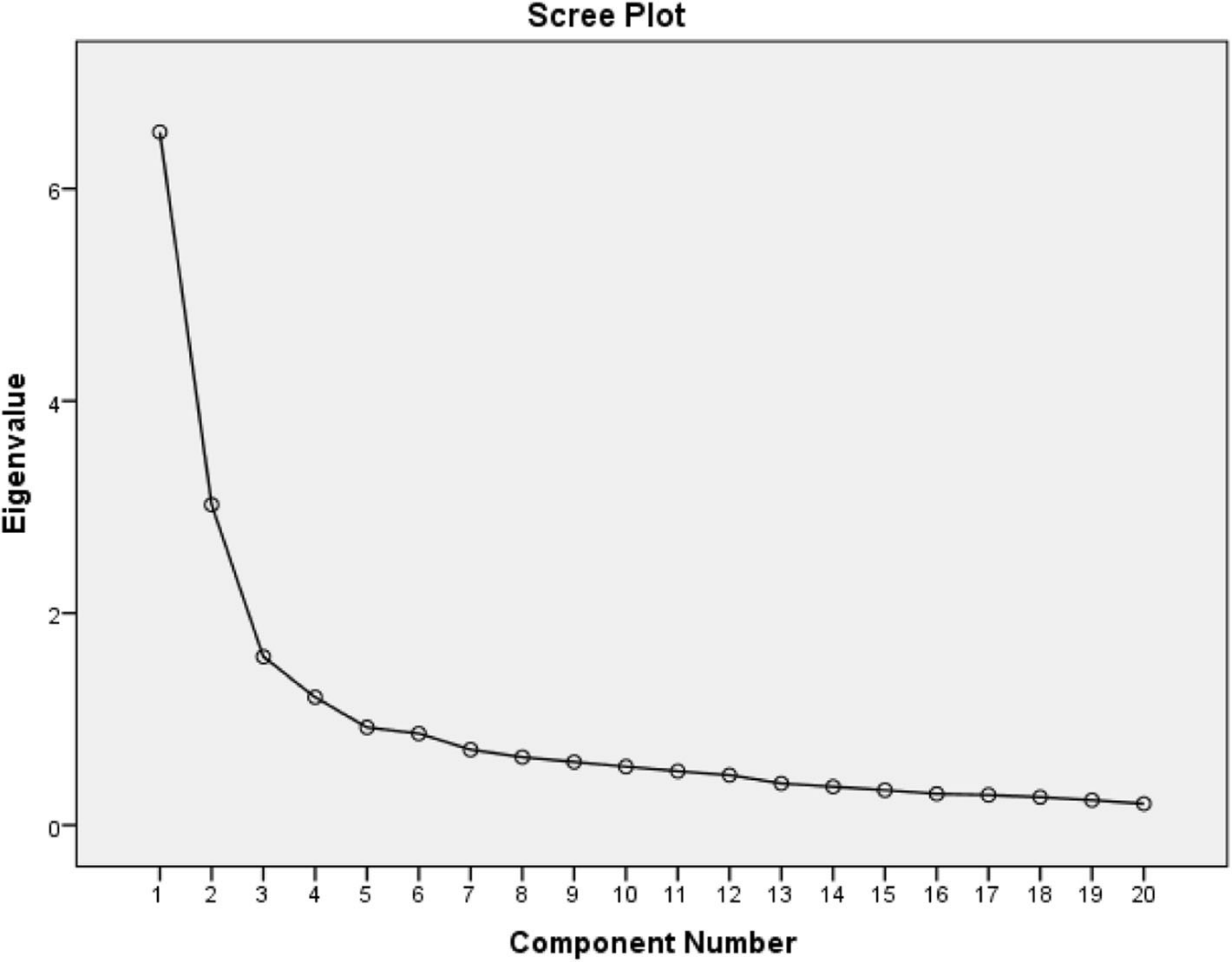
A scree plot showing the number of components to retain for further investigation

Scree plot was used to ascertain the number of appropriate components (factors) for the instrument. The scree plot of all the items showed a clear break between the second and third component (Fig. 1) which signifies that two (2) components explained most of the variance. Additionally, the component matrix (Kaiser Criterion with eigenvalues > 1) shows most of the items loading strongly on two components; this further confirmed that a two factor solution is more appropriate. Therefore, a two-component solution was performed using Oblimin rotation and this explained a total of 47.8% of the variance, with component 1 contributing 32.7% and component 2 contributing 15.1% of the variance. Table 2 shows the structured loading of items on the two components as revealed by the rotated solution.

**Table 2:** Oblimin rotation and loading of items on two components

Pattern Matrix		
	Component	
	1	2
Item 10	0.743	
Item 8	0.738	
Item 9	0.711	
Item 11	0.710	
Item 5	0.695	
Item 2	0.683	
Item 4	0.681	
Item 12	0.679	
Item 1	0.669	
Item 13	0.641	
Item 3	0.591	
Item 7	0.563	
Item 6	0.509	
Item 17		0.801
Item 15		0.779
Item 18		0.768
Item 16		0.745
Item 20		0.661
Item 14		0.634
Item 19		0.630

### Mean scores for self-reported intercultural competence and institutional inclusiveness

Table 3 shows the mean responses to each of the items on the instrument and across each domain. Highest mean scores were obtained in four domains - respondents indicated that they are prepared to adjust their cultural knowledge during interaction with people from other cultures (M = 4.56) and also enjoyed interacting with people from different culture (M = 4.53). They agreed to having awareness about the role of culture in their field of study (M = 4.39) and are confident participating in multicultural groups (M = 4.44). Generally the students tend to agree that their intercultural competence had been developed through the medical program (M = 4.22). Concerning institutional approach to fostering intercultural inclusiveness, respondents indicated a strong agreement to the presence of respect of cultural differences within their learning institution (M = 4.40). The lowest mean scores in this section were obtained in relation to the ability of teachers to understand the needs of international students (M = 3.62), and efforts made by teachers to help international students (M = 3.44). Overall, respondents had significantly lower ratings (*p* < 0.000) for institutional intercultural inclusiveness (M = 4.07) in comparison to their self-reported intercultural competence (M = 4.22).

**Table 3:** Mean scores of the survey items

Statement	Mean	SD
I have a great awareness of cultural diversity	4.14	0.79
I have a good understanding of cross-cultural interpersonal skills	3.78	0.82
I am aware of the role of culture in my chosen field of study	4.39	0.74
I am conscious of the cultural knowledge I use when interacting with people with different cultural backgrounds	4.05	0.75
I am conscious of the cultural knowledge I apply to cross-cultural interactions	3.95	0.77
I am prepared to adjust my cultural knowledge as I interact with people from an unfamiliar culture	4.56	0.69
I enjoy living in cultures that are unfamiliar to me	3.93	1.01
I am confident that I could socialise with locals in an culture that is unfamiliar	3.98	0.88
I am certain that I could deal better with adjusting to a culture that is new to me	3.89	0.89
I am confident with communicating with people from culturally different backgrounds	4.07	0.79
I am able to make social contact with culturally different others	4.19	0.78
I am confident participating in multicultural groups	4.44	0.73
I enjoy interacting with people from different cultures	4.53	0.69
General self-reported intercultural competence (13 Items)	4.20	0.64
My teachers encourage contact between students from different cultural backgrounds	3.91	0.96
My teachers make special efforts to help international students	3.44	1.05
Cultural differences are respected in my university	4.40	0.73
My teachers understand the needs of international students	3.62	0.99
In my class, there are opportunities for student to learn about different cultures	3.79	0.96
My classmates are accepting of cultural differences	4.17	0.79
Students from different cultural groups works well with each other in my classes	4.20	0.82
Institutional intercultural awareness and inclusiveness (7 Items)	3.90	0.78
All items (20 items)	4.07	0.47

### Association of demographic characteristics with responses to survey items

Participants’ responses to survey items on self-reported intercultural competence was only affected by rurality, in which students from major cities rated themselves higher in terms of their intercultural competence levels in comparison to those from rural settings (*p* = 0.008; Median score = 4.20 vs 3.99). However, responses to institutional inclusiveness survey items were significantly affected by age-group, ethnic origin and time lived in Australia (Table 4). Younger students (≤20 years) gave significantly higher (*p* = 0.002) mean scores (Median score = 4.0) for institutional inclusiveness than their older counterparts (Median score = 3.78). Similarly, participants of Australian ethnic origin gave higher ratings (Median score = 4.0; *p* = 0.001) than participants from other continents (Median score range = 3.46–3.90) and those who had stayed longer in Australia gave higher ratings (Median score range = 4.0–4.14; *p* = 0.000) than their recently arrived counterparts (Median score range = 3.57–3.64). Furthermore, there was year of study effect in which the 2014 respondents gave significantly lower mean scores (M = 3.23–4.20) across the seven (7) items in the institutional intercultural inclusiveness domain in comparison to all the other study year groups (M = 3.41–4.50; *p* < 0.002).

**Table 4:** Demographic variables’ effect on institutional inclusiveness survey items

Variables	Categories	Median (IQR)	Test Statistic (df)	*p*-value
Gender	Male (172)	4.0 (0.86)	1.08 (1))	0.299^NS^
	Female (323)	4.0 (0.86)		
Age group	≤20 (373)	4.0 (0.86)	9.66 (1)	0.002^**^
	> 20 (142)	3.78 (1.0)		
Ethnic Origin	Africa (40)	3.78 (0.96)	18.75 (4)	0.001^**^
	Asia (135)	3.85 (0.86)		
	Australia (290)	4.0 (0.71)		
	Europe (22)	3.90 (0.89)		
	America (22)	3.46 (1.32)		
Rurality/Town	Rural (160)	4.0 (0.86)	3.07 (2)	0.215^NS^
	Regional (152)	4.0 (0.86)		
	City (197)	3.85 (0.86)		
Time lived in Australia	< 1 yr. (73)	3.57 (1.14)	23.48 (5)	0.000^***^
	1-5 yrs. (40)	3.64 (0.96)		
	6-10 yrs. (51)	4.0 (0.86)		
	11-15 yrs. (37)	4.0 (1.29		
	> 15 yrs. (28)	4.14 (1.25)		
	Born in Australia (286)	4.0 (0.71		
Study year	2014 (137)	3.71 (0.93)	15.25 (3)	0.002^**^
	2015 (105)	4.0 (0.79)		
	2016 (140)	4.0 (0.86)		
	2017 (133)	4.0 (0.71)		

*NS* not significant, **p < 0.01, ***p < 0.001

## Discussion

Our study examined first year medical students’ awareness of their own intercultural competence levels and their perceptions of intercultural inclusiveness within our institution, using a self-reported survey tool. All domains in the survey instrument showed excellent internal consistency. The present result on the domain relating to general cross-cultural interpersonal competence correlates with similar high findings of Cronbach’s alpha = 0.95 in another study [34]. The results of the factor analysis shows a simple structure pattern with all items in the intercultural competence domain loading strongly on component 1 and the institutional inclusiveness domain items loading strongly on component 2. This shows that all items fit well with other items in the domain or component, and are all valid and useful in assessing the study objectives.

Generally, the students reported high intercultural competence levels in the first 6 months of their learning of the medical curricula. Majority agreed that the program fostered their awareness of cultural diversity, good understanding of cross-cultural interpersonal skills and prepared them to adjust their cultural knowledge as they interact with people from different cultural backgrounds, echoing the results of Knott et al. [34] and Jacobs et al. [35]. As noted by Dunstan [36], social integration is a critical factor in supporting successful and satisfying learning experiences [36]. Additionally, majority of the students strongly agreed that the medical program improved their awareness of the role of culture in medicine, confirming the results from another study [37]. Since self-efficacy is a necessary antecedent in the further development of intercultural competences [38]; these findings are indicators of readiness of participants to integrate intercultural skills into their career.

The confidence of respondents about their ability to communicate and interact with people from different cultural backgrounds may or may not have totally resulted from the impact of the medical program since attaining efficient communication skills usually requires some time. It may be that students already have developed intercultural communication skills due to their previous educational experiences prior to entry into the medical program. The higher rating of students who had lived in major cities on their intercultural competence level confirms this because intercultural competence is strongly influenced by cultural identity sources such as the living environment. Experience of life in major cities - which likely will be composed of diverse cultures, may provide the exposure to various social events that invariably predispose people to improved development of intercultural awareness, sensitivity and communication skills that may not be easily acquired in environments with smaller population density [39]. It is therefore imperative that baseline data on intercultural competence skills are collected prior to commencing medical degree to aid faculty in ascertaining students’ baseline level of intercultural competence and to evaluate the effectiveness of the program in training them in this area.

Overall, the students reported good level of intercultural inclusiveness by the educational institution; where majority of them agreed that cultural differences are respected in the institution and that students from diverse cultural groups work well with each other in their classes. Conversely, many students were not too pleased with the effort their teachers were making to help or understand the needs of international students. They also felt that there were not enough opportunities provided for students to learn about different cultures. Furthermore, the lower mean scores from international students on institutional intercultural inclusiveness is similar to the findings of Mak et al. [28] who indicated that cultural distance (differences in values and communication styles) is a significant predictor of acceptance and adjustment of international students [28]. Nevertheless, this finding merits serious consideration, as international students constitute a substantial proportion of the medical students in the institution. Additionally, older students’ experiences of lesser sense of cultural inclusiveness may suggest that this group of students have more difficulty in transitioning into University life [40].

Our findings highlight a major area for improvement in which faculty members need to be trained to better manage diversity and proactively engage in internationalisation of student outlooks. Various researchers have emphasised the important role of teachers in facilitating intercultural interaction and competence among their students [41–44]. Freeman et al. [45] advocated a “community of practice” approach which embeds inclusive teaching practices and intercultural competence development in the formal curriculum, and evaluates the subsequent impact on student outcomes.

Further exploration via qualitative research will provide more insights into strategies that could be employed to foster increasing intercultural inclusiveness. Future research studies may examine the specific areas of help international students might require of their teachers to have a sense of belonging in the academic system. Teachers’ support for international students may include clarity of instructions as other studies have highlighted that international students in Australia have considerable difficulty with the Australian accent [46–48]. According to Kift [49], transitions for students who commence a journey of higher degree in an overseas country, requires a scaffolding support [49]; therefore considerations of specific needs of international students might help to lessen difficulties encountered by this group of students.

The observed higher mean scores from respondents in the later years (2015, 2016, and 2017) in comparison to the respondents in the year 2014 may be indicative of improvement in the level of institutional intercultural inclusiveness, which is the inclusion of more intercultural teaching and learning programs in the curriculum over time. Such programs included the effects of racism on social and physical health and emphasis on global medicine with a specific focus on refugee healthcare. This suggests more favorable intercultural experiences for students enrolled in the medical program from 2015 to 2017, although, the conclusion that can be drawn is somewhat limited by lack of comparative data from students in other disciplines. It would be advantageous for medical educators to regularly examine their students’ perceived level of intercultural competence and satisfaction with the institutions’ cultural inclusiveness practices and curricula, longitudinally and cross-sectionally, to evaluate the effectiveness of the curricula and remediate teaching areas in need of improvement. Any gap found may inform professional development for staff on ensuring strong intercultural inclusiveness in their activities with students. Furthermore, it could serve as a diagnostic tool for identifying students’ learning needs in the area of intercultural competence.

### Strengths and limitations of the study

The main strength of this paper is that it is a comprehensive four-year study that examined first year medical students’ perceptions of intercultural competence. This approach allowed for identification of curriculum gaps in relation to cultural diversity. However, the findings of the study are limited by its use of a self-assessment method which is subjective and influenced by beliefs and values that individuals hold about themselves. Self-evaluation instruments may not be able to judge the accuracy of an individual’s evaluation of their own deficits [50]. Additionally, this study did not include participants’ practical demonstration of intercultural competence. However, self-awareness of an individual’s intercultural competence level is a crucial first step that triggers appropriate behavioural adjustments to cultural differences. Moreover, since the students were still in the first year of their study and not yet in their clinical years, at this stage, the use of survey to assess their intercultural competence level is sufficient to reveal their learning needs. Furthermore, given that the students were exposed to interactive learning sessions, which allowed for robust discussions and reflections on intercultural competence, they would have understood and recognised their individual deficits. This could facilitate the evaluation of the curriculum by the faculty with clearer and more explicit inclusion of teaching on intercultural competence in order to adequately prepare students to be able to practice health care delivery in culturally diverse contexts. Future research could consider qualitative exploration of how intercultural competence can be fostered among educators and students.

## Conclusion

It is important for medical educators to regularly examine their students’ perceived level of intercultural competence and satisfaction with institutional cultural inclusiveness. This will enhance effective evaluation of the curriculum and identification of teaching areas that are in need of improvement. Ultimately, this process could help to ensure that medical students are well prepared to provide culturally sensitive and effective patient-centered care in their medical career.

## Data Availability

All data generated and analysed during this study are included in this published article.

## Abbreviations

AMC: Australian Medical Council
HE: Australian Higher Education
JCU: James Cook University
